# Reducing teachers’ perceived stress through an online wellbeing intervention: the role of socio-emotional competence and self-efficacy

**DOI:** 10.3389/fpsyg.2025.1726492

**Published:** 2026-01-09

**Authors:** Veronica Ornaghi, Valeria Cavioni, Elisabetta Conte

**Affiliations:** 1 Department of Human Sciences for Education “Riccardo Massa”, University of Milano-Bicocca, Milan, Italy; 2 Department of Humanities and Social Sciences, Faculty of Society and Communication Sciences, Universitas Mercatorum, Rome, Italy; 3 Department of Human and Social Sciences, University of Bergamo, Bergamo, Italy

**Keywords:** teachers, perceived stress, socio-emotional competence, self-efficacy, evidence-based intervention, teacher wellbeing, online training

## Abstract

**Introduction:**

Teaching is a profession characterized by high emotional demands and exposure to multiple stressors, often leading to elevated levels of perceived stress. Self-efficacy and socio-emotional competence have been identified as key protective resources that enable teachers to cope more effectively with professional challenges. The present quasi-experimental study evaluated the effectiveness of an Online Wellbeing Course (OWC) in enhancing teachers’ socio-emotional competence and self-efficacy and reducing perceived stress.

**Methods:**

A total of 151 in-service Italian teachers took part in the study, with schools randomly assigned to an experimental group (OWC; *n* = 72) or a control group (*n* = 79). Participants completed validated measures of perceived stress, socio-emotional competence, and self-efficacy at pre- and post-test.

**Results:**

Teachers in the OWC group reported significant improvements in socio-emotional competence and self-efficacy, as well as a reduction in perceived stress compared to the control group. Moreover, pre- to post-test gains in self-efficacy - but not socio-emotional competence - significantly mediated the relationship between participation in the OWC and reductions in perceived stress.

**Discussion:**

These findings provide empirical evidence for the effectiveness of online wellbeing interventions in supporting teachers’ psychological resources and reducing stress. Implications for teacher professional development are discussed.

## 1 Introduction

The teaching profession entails considerable emotional labor and has been consistently associated with elevated levels of stress and burnout (Kariou et al., 2021). Educators are frequently confronted with a range of stressors, including excessive workload, role ambiguity, insufficient institutional support, classroom management challenges, bureaucratic demands, and other occupational difficulties (Mérida-López and Extremera, 2017; Mijakoski et al., 2022). Consequently, sustained exposure to these work-related stressors has been shown to place teachers at heightened risk for developing anxiety and stress (Cooper and Travers, 2012).

In the face of the many actual stressors that characterize the teaching profession, what seems crucial is how teachers perceive and feel about their lack of control and unpredictability. Beyond objective stressors, what often determines wellbeing outcomes is teachers’ subjective appraisal of their resources relative to demands, captured by the concept of perceived stress.

Perceived stress has been documented to be associated with a number of teachers’ individual factors, such as socio-emotional competence and self-efficacy (Jennings and Greenberg, 2009; Schwarzer and Hallum, 2008; Zee and Koomen, 2016). A recent meta-analysis confirmed that personal resources such as self-efficacy, socio-emotional competences, and autonomous motivation are among the strongest predictors of teacher wellbeing across large international samples (Zhou et al., 2024). However, perceived stress is not only influenced by individual resources but also by contextual and organizational dynamics. In this regard, Zagni et al. (2025) showed that in the Italian school system, risk factors such as workload, role conflict, and low perceived support tend to accumulate, thereby increasing teachers’ stress levels. At the same time, protective resources—including self-efficacy and socio-emotional competence—significantly mitigate these negative effects. More specifically, these psychological resources act as protective mechanisms that help educators reinterpret stressful events, regulate emotions, and sustain motivation in the face of adversity. Training programs that explicitly target the development of socio-emotional competences and efficacy beliefs have therefore become a priority in teacher professional development, as they enable teachers to cope more effectively with challenging situations while maintaining high levels of professional functioning (Cavioni, 2025; Collie et al., 2012; Mansfield et al., 2016).

Following the COVID-19 pandemic, teacher training practices underwent profound changes, with a shift toward digital and distance-based formats. This transformation highlighted both new opportunities and challenges. In fact, while online training increased accessibility and flexibility, it also required innovative approaches to maintain engagement and effectiveness in fostering wellbeing. Building on these considerations, the present study examines the role of socio-emotional competence and self-efficacy in protecting against perceived stress and evaluates the potential of online wellbeing programs to enhance these resources.

### 1.1 Teachers perceived stress in the school context

Perceived stress is an individual’s subjective judgment of the factors that contribute to being stressed (Kyriacou, 2011). In particular, a person experiences high levels of stress when the demands of the situation are seen as being excessive as compared to the available resources and coping skills.

In the school context, this dynamic is particularly evident, as it exposes teachers to constant interpersonal interactions, performance expectations, and institutional pressures (Collie et al., 2017; Kyriacou, 2001). Teaching has repeatedly been identified as one of the most stressful occupations, since teachers not only manage academic instruction but also bear responsibility for students’ behavior, emotional needs, and social development (Nwoko et al., 2023; Travers and Cooper, 1996). Consequently, the school context can be considered a potentially stressful setting in which the combination of heavy workload, classroom management difficulties, accountability requirements, and limited resources may overwhelm teachers’ coping capacities, thereby increasing their perceived stress.

How teachers perceive stress is related to their level of wellbeing. In fact, high levels of perceived stress are significantly associated with the risk of burnout (Bottiani et al., 2019; McCormick and Barnett, 2011; Nwoko et al., 2023), which is defined as a prolonged response of a person chronically exposed to emotional and interpersonal stressors in the workplace (Maslach et al., 2001). The consequences of experiencing burnout can be very severe. Research has shown that individuals who experience burnout tend to report feelings of being helpless, hopeless, and powerless. As such, these persons are more likely to develop symptoms of depression due to the consequences of the sustained and uncontrollable stress and emotional exhaustion experienced (Agyapong et al., 2022; Bianchi and Schonfeld, 2016; Capone et al., 2019; Schaufeli and Buunk, 2004). The prolonged difficulties of effectively managing one’s environment and personal tasks to be able to actively tackle stressors is a key factor in established theories related to mental health diseases, such as depression (e.g., Peterson et al., 1993).

The effect of perceived stress on teachers’ wellbeing is amplified in difficult and challenging situations, as emerged world-wide during the COVID-19 pandemic (Katsarou et al., 2023; Levante et al., 2023; Lizana et al., 2021; Pellerone, 2021; Sacré et al., 2023). The critical situation and increasingly challenging demands faced by teachers during the pandemic (lock-down, social distancing, use of remote instruction formats, etc.) veritably increased stress factors, levels of anxiety, and the incidence of burnout, which was found to be related to the prevalence of negative emotions and emotional dysregulation (Carroll et al., 2022). Geraci et al. (2023) found that the pandemic emergency did indeed increase the risk of teachers’ burnout and negatively impacted their level of work engagement, which is a persistent, positive and satisfying work-related mental state, characterized by vigor, absorption and dedication to work activities. A recent study by Conte et al. (2024) likewise showed that adverse working conditions, lack of support, precariousness, and a demanding school system were key stressors after the pandemic, while emotion-focused coping strategies played a crucial protective role for teachers’ wellbeing.

Research also highlights that perceived stress is related to teachers’ years of experience, even if their association is complex and non-linear. Indeed, early-career teachers tend to report high stress levels, mainly due to classroom management difficulties, lack of professional recognition and of established coping strategies (Brewer and Shapard, 2004; Carroll et al., 2022; Gavish and Friedman, 2010), whereas more experienced teachers often benefit from greater resilience and consolidated resources (Zhou et al., 2024). However, an extended teaching experience does not automatically protect against stress, as veteran teachers may still be exposed to high levels of burnout, bureaucratic overload, and systemic changes (Skaalvik and Skaalvik, 2017; Van Droogenbroeck et al., 2014). This pattern is consistent with evidence showing that novice teachers tend to experience higher acute stress, while more experienced teachers may face cumulative burnout-related risks (Gooden et al., 2023).

### 1.2 Socio-emotional competences and self-efficacy as protective resources

Socio-emotional competences are increasingly recognized as critical psychological resources that enable teachers to manage the demands of their profession (Nwoko et al., 2023). These skills encompass self-awareness, emotion regulation, empathy, constructive relationship-building, and responsible decision-making (Durlak et al., 2025). Such abilities facilitate more effective interpretation and regulation of emotional experiences, thereby reducing feelings of overload when encountering professional challenges. Stress, conceptualized as a mismatch between environmental demands and available coping resources, has long been identified as a key antecedent of burnout, which manifests in emotional exhaustion, depersonalization, and diminished professional efficacy (Maslach and Jackson, 1981). Socio-emotional competences mitigate this process by promoting adaptive coping responses, sustaining positive relationships with students and colleagues, and fostering a supportive classroom climate (Jennings and Greenberg, 2009). Emotion regulation skills, in particular, are central in preventing the escalation of negative affect into chronic stress and subsequent exhaustion (Brackett, 2019; Brackett et al., 2011; Cervellione et al., 2025). Furthermore, research on achievement emotions has shown that teachers’ capacity to manage both positive and negative emotions shapes their motivation, instructional performance, and long-term psychological health (Pekrun, 2006, 2024).

Empirical evidence supports these theoretical claims. Ornaghi et al. (2023) demonstrated that teachers with stronger socio-emotional competences reported lower burnout levels, primarily because these competences enhanced work engagement, thereby attenuating the adverse impact of perceived stress. Similarly, Conte et al. (2025) found that socio-emotional competences longitudinally predicted reduced stress in the short term and lower burnout over time, even after controlling for teaching experience. Collectively, these findings suggest that socio-emotional competences protect teachers not only from the immediate consequences of stress but also promote resilience and sustained wellbeing throughout the school year.

Parallel to socio-emotional competences, teachers’ sense of self-efficacy - defined as beliefs in their ability to effectively organize and execute instructional practices (Skaalvik and Skaalvik, 2007)—has emerged as a robust protective factor against stress. The protective function of teachers’ self-efficacy is strongly supported by recent findings showing that work-related self-efficacy is associated with lower perceived stress and higher job satisfaction (Jentsch et al., 2023). Higher levels of self-efficacy are associated with improved classroom management, stronger student motivation (Hettinger et al., 2024), and greater perseverance in the face of challenges, all of which contribute to reduced stress and prevention of emotional exhaustion (Zee and Koomen, 2016). Conversely, low self-efficacy undermines teachers’ capacity to cope with professional demands, thereby increasing vulnerability to burnout and disengagement. Teachers with a strong sense of efficacy are more likely to adopt proactive coping strategies, employ effective problem-solving, and sustain motivation even under pressure. This confidence not only reduces stress perception but also supports perseverance, nurtures positive classroom climates, and lowers the risk of burnout (Aloe et al., 2014). Importantly, self-efficacy functions as a protective barrier between occupational stressors and psychological strain, underscoring its central role in teacher resilience. Promoting teachers’ self-efficacy beliefs is therefore essential not only for safeguarding their wellbeing but also for maintaining educational quality, as efficacious teachers are better positioned to establish supportive relationships and serve as models of adaptive coping for their students (Cavioni, 2025; Cavioni et al., 2023b).

### 1.3 Online teachers’ training

Targeted professional development programs that enhance socio-emotional competence and self-efficacy have been shown to reduce stress and promote teacher wellbeing (Berg et al., 2021; Cavioni et al., 2023a; Mansfield, 2020). The shift toward online training after the pandemic has expanded the accessibility of such interventions, while gamified approaches further enhance motivation and engagement (Liu et al., 2023). Despite these promising developments, empirical evidence on the effectiveness of online wellbeing programs for teachers remains scarce, particularly with respect to the mechanisms through which they reduce stress.

With regard to serious games, they have been described as interactive digital environments that provide learners with realistic simulations of complex situations, thereby fostering active, engaging, and participatory learning (Kapp, 2012; Swacha, 2021). A key strength lies in the possibility to make mistakes without the negative emotions usually associated with real-life problem solving—such as anxiety or frustration—thus supporting experimentation and resilience (Dalgarno and Lee, 2010). Moreover, the integration of storytelling and immersive challenges has been shown to elicit emotions like joy, curiosity, and interest, which in turn enhance motivation and persistence (Shute et al., 2015a, 2015b; Rooney, 2012). Finally, gamification elements such as rewards and progress tracking strengthen learners’ sense of achievement, reinforcing the transfer of skills from the virtual context to professional practice (Kalogiannakis et al., 2021; Seaborn and Fels, 2015; Sailer et al., 2017).

Despite the growing use of digital training and serious games in education, most initiatives have been designed for students with gamified approaches already applied to enhance learning or reduce behavioral problems such as bullying and cyberbullying (e.g., Martel-Santana and Martín-del-Pozo, 2025). By contrast, existing tools rarely address teachers’ professional wellbeing. This gap highlights the need for innovative online and gamified training that can directly support educators’ social–emotional skills, their sense of efficacy, and their occupational wellbeing. In addition, contemporary research on serious-game-based interventions specifically targeting teachers remains extremely limited. This scarcity of recent empirical evidence further underscores the novelty and relevance of the present study, which offers quantitative data to an emerging and still largely unexplored area of investigation.

Qualitative evidence has already highlighted the potential of a game-based Online Wellbeing Course (OWC) to foster teachers’ emotional awareness, stress management strategies, and relational skills (Cavioni et al., 2024; Cavioni, 2025). Teachers reported perceived benefits in terms of coping strategies, collegial collaboration, and positive school climate. However, these insights were based on self-reported experiences collected through focus groups, and no quantitative assessment of effectiveness was carried out. In this work we address this gap by providing experimental evidence on the OWC’s impact using validated measures of stress, socio-emotional competence, and self-efficacy.

### 1.4 The present study

Building on previous qualitative findings (Cavioni et al., 2024), this study adopts a quasi-experimental design to explore the potential effects of the OWC using standardized instruments. This approach allows us to move from exploratory accounts of teachers’ experiences toward a more rigorous, evidence-based assessment of the effectiveness of the intervention.

The present study aimed at investigating whether participation in the OWC may be associated with changes in in-service teachers’ socio-emotional competence, self-efficacy, and perceived stress, while also controlling for teachers’ job experience. Specifically, we expected that (H1) teachers in the Experimental Group would show greater improvements in socio-emotional competence and self-efficacy compared to those in a Control Group; and (H2) that these improvements might mediate reductions in perceived stress. By examining these associations, the study seeks to provide preliminary evidence on the role of gamified training for teachers’ wellbeing.

## 2 Materials and methods

### 2.1 Participants

A total of 151 (138 females; mean age: 45.5 ± 8.8 years) in-service Italian teachers took part in the study. They were recruited from 10 schools - kindergartens and primary schools (50.3%), middle and high schools (49.7%) - in both urban and rural areas of Northern Italy. The teachers had an average of 16.6 years of teaching experience (*SD* = 11.1). Ninety-four percent of them were employed full time. Prior to participating in the study, all individuals were told about the research objectives and signed an informed consent form. The inclusion criteria were as follows: (1) working as an in-service teacher and (2) agreeing to the terms of participation in the study. There were no exclusion criteria.

The study was carried out in compliance with the American Psychological Association’s ethical principles and code of conduct and received the approval of the University of Milano-Bicocca Ethics Committee (protocol number: 0129650/21). Participants were free to withdraw from the study at any time and received no monetary or other financial reward. There were no conflicts of interest among the authors in relation to the research.

### 2.2 Measures and procedure

In order to assess teachers’ perceived stress, socio-emotional competence, and self-efficacy, participants were asked to complete the following self-report validated measures.

The *Perceived Stress Scale* (PSS; Cohen et al., 1983) is a well-known psychological instrument that was developed with the intention of determining the degree to which individuals view their own lives as being stressful. The scale is comprised of 10 items, each of which is a statement that the participant is asked to rate on a Likert-type response scale from 0 (never true) to 4 (very often true). The purpose of the statements is to determine the degree to which a person has experienced specific sensations and ideas associated with stress. The Italian version of the scale was used in this study (Fossati, 2010). Reliability coefficients of the overall scale were Cronbach’s α = 0.82 at pre-test and Cronbach’s α = 0.84 at post-test.

The *Social and Emotional Competence of Teachers questionnaire* (SECTRS; Tom, 2012), which consists of 25 items that measure four areas: Teacher-Student Relationships (interactions between teachers and students; sample item: “I am good at understanding how my students’ feel”), Emotion Regulation (teachers’ ability to manage their emotions in challenging situations; sample item: “I remain calm when addressing student misbehavior”), Social Awareness (teachers’ sensitivity to diversity and awareness how their practice impacts students: sample item: “I consider my students’ wellbeing when making decisions”), and Interpersonal Relationships (teachers’ relationships with parents and school staff; sample item: “In conflict situations with staff members, I can effectively negotiate solutions”). Teachers were asked to express their agreement or disagreement with the items on a 6-point Likert scale, from 1 (strongly disagree) to 6 (strongly agree). The Italian validated version of the questionnaire was adopted (Grazzani et al., 2024). In the original instrument, Cronbach’s alpha coefficients for the four subscales ranged between 0.69 and 0.81 (Tom, 2012). In our sample, reliability coefficients ranged from 0.74 to 0.81 at pre-test and from 0.73 to 0.80 at post-test, replicating the good internal consistency reported in the original validation.

The *Norwegian Teacher’s Self Efficacy Scale* (NTSES; Skaalvik and Skaalvik, 2007) is a 24-item questionnaire evaluating teachers’ different dimensions of self-efficacy on a 7-point response scale ranging from 1 (*not certain at all*) to 7 (*absolutely certain*). The items are grouped in the following subscales (4 items each): Instruction (sample item: “How certain are you that you can answer students’ questions so that they understand difficult problems?”); Adapt instruction to individual needs (sample item: “How certain are you that you can organize schoolwork to adapt instruction and assignment to individual needs”); Motivate students (sample item: “How certain are you that you can motivate students who show low interest in schoolwork”); Maintain discipline (sample item: “How certain are you that you can get students with behavioral problems to follow classroom rules”); Cooperate with colleagues and parents (sample item: “How certain are you that you can cooperate well with most parents”); Cope with change (sample item: “How certain are you that you can successfully use any instructional method that the school decides to use?”). Participants were administered the Italian validated version of the NTSES (Avanzi et al., 2013). Reliability coefficients of the overall scale were Cronbach’s α = 0.94 at pre-test, and Cronbach’s α = 0.96 at post-test and reliability coefficients of the subscales at pre- and post-test ranged between Cronbach’s α = 0.85 and Cronbach’s α = 0.95, confirming the good psychometric properties of the instrument in our sample.

Finally, *socio-demographic data* were also collected in terms of age, gender, and years of teaching experience. Data were collected in October 2022 (pre-test) and May 2023 (post-test).

Before completing the online questionnaires, during a face-to-face meeting, teachers were informed about the research and were asked to provide their consent to participate in the study.

### 2.3 Research design and intervention

During the academic year 2022/2023, all participants were pre- and post-tested with the above described measures. Schools were randomly assigned to the conditions and the distribution of teachers in each group was as follows: *N* = 72 in the experimental group, and *N* = 79 in the control group. Teachers in the experimental group received the OWC training intervention immediately after the pre-test, while those in the control group were granted access only after the post-test. The intervention was delivered fully online over the five-month period between pre- and post-test. The OWC consisted of 12 structured levels released at the rate of one level per week, guiding teachers through a progressive sequence of gamified activities and reflective tasks. Teachers in the EG were given individual access to the OWC platform and were invited to follow the weekly release of levels at their own pace, engaging with the interactive scenarios and the accompanying digital handbook. To sustain participation and support continuity, the research team sent regular email reminders and invitations to proceed to the next level. The course was entirely self-paced and asynchronous, and the platform automatically recorded participants’ progress across all levels. Apart from technical support, no additional structured follow-up meetings were organized; teachers in both groups continued their usual school activities throughout the study. The OWC is a serious game designed within the European project *Teaching to Be: Supporting Teachers’ Professional Growth and Wellbeing in the Field of Social and Emotional Learning* to support teachers’ professional wellbeing.^1^ The intervention, delivered over 5 months, combined gamified scenarios, reflective prompts, and a supplementary handbook. Its design integrated evidence from the literature on teachers’ risk and protective factors with a participatory action research approach, actively involving teachers in refining its contents. The OWC incorporated immersive and interactive scenarios, narrative storytelling with teacher avatars, reflection prompts, quizzes, and progress monitoring systems. Participants could collect virtual items, earn “extra wellbeing points,” and track their development through gamified feedback and rewards. The game was complemented by a digital handbook including self-assessments, journaling, and goal-setting activities, aimed at deepening reflection and facilitating offline peer discussions. Topics across levels included stress regulation, classroom management, empathy, self-care, leadership, and decision-making, with the overarching goal of supporting personal wellbeing and fostering a positive school climate (for a complete description of the course and its contents see Cavioni et al., 2024).

### 2.4 Overview of data analysis

All statistical analyses were conducted using IBM SPSS Version 29. Teachers’ data were matched by code to combine the pre- and post-test scores. We assessed the distribution of data for each of the study measures. Normal distribution of scores was confirmed, with no variables exhibiting kurtosis or skewness beyond recommended limits of −2 and +2 (George and Mallery, 2010). We also verified homogeneity of variances with Levene’s tests, which were non-significant.

Next, the main descriptive statistics and zero-order correlations were computed. With the aim of verifying the equivalence of the three groups prior to the intervention, a series of analyses of variance (ANOVAs) were run to compare teachers’ performances at pre-test as a function of group condition. To verify the impact of the intervention on teachers’ emotional competence, resilience, self-efficacy, and level of perceived stress, we used a General Linear Model (GLM) for repeated measures. The independent variables were Time (pre-test and post-test) as a within-subject factor and Group Condition (OWC and control group) as a between-subject factor. The dependent variables measured at two-time points were teachers’ scores of perceived stress, socio-emotional competence, and self-efficacy. We included years of teaching experience as covariate. Effect sizes were calculated using partial eta squared (*ր_p_*^2^) values.

After verifying the overall effect of the intervention on all variables under investigation, we examined whether improvements in socio-emotional competence and self-efficacy accounted for reductions in perceived stress by conducting parallel multiple mediation analyses. The independent variable was Group condition (0 = control, 1 = OWC). The mediators were change scores from pre- to post-test in SEC (ΔSEC) and SE (ΔSE), calculated by subtracting pre-test scores from post-test scores. The dependent variable was change in perceived stress (ΔPSS). Indirect effects were estimated using bias-corrected bootstrapping with 5,000 resamples. The variable years of teaching experience was included as covariate.

## 3 Results

### 3.1 Descriptive and preliminary analyses

Table 1 shows the zero-order correlations and descriptive statistics for all the study variables considering the whole sample. Significant correlations emerged between perceived stress, socio-emotional competence, and self-efficacy at both pre- and post-test stages. Means and standard deviations of all measures by group condition at two time points (pre and post-test) are reported in Table 2.

**Table 1:** Zero-order correlations and descriptive statistics of the study variables (*N* = 151).

	1	2	3	4	5	6	7
Years of teaching experience	–						
Perceived Stress (PRE)	0.022	–					
Emotional Competence (PRE)	−0.126	−0.352**	–				
Self-Efficacy (PRE)	0.078	−0.410**	0.500**	–			
Perceived Stress (POST)	−0.188*	0.448**	−0.089	−0.074	–		
Emotional Competence (POST)	0.089	−0.378**	0.621**	0.456**	−0.359**	–	
Self-Efficacy (POST)	0.138	−0.301**	0.309**	0.457**	−0.367**	0.637**	–
Mean	16.580	17.364	118.185	103.053	15.920	119.457	106.013
Standard deviation	10.497	6.389	12.131	14.720	6.330	12.491	16.628
Kurtosis	0.533	0.027	−0.521	−0.261	0.303	−0.584	−0.777
Skewness	−0.678	−0.598	0.551	−0.490	−0.091	0.768	0.969

*p < 0.01, **p < 0.001. Variable numbers in columns correspond to the variables listed in the rows.

**Table 2:** Pre- and post-test means and standard deviations for all variables by group condition (OWC vs Control).

	OWC group		Control group	
	Pretest	Post-test	Pretest	Post-test
Perceived stress	17.944 (6.270)	14.819 (5.974)	16.835 (6.489)	16.924 (5.974)
Socio-emotional competence	116.486 (11.930)	120.263 (10.891)	119.734 (12.181)	118.721 (13.818)
Self-efficacy	100.305 (15.538)	106.902 (16.133)	105.557 (13.550)	105.202 (17.128)

Standard deviations are reported in brackets.

To compare teachers’ performance at pre-test by group condition, a series of ANOVAs were run. No significant difference emerged concerning the administered measures, with the exception of the self-efficacy scale, on which teachers in the control group obtained higher scores than teachers in the OWC group: PSS, *F*(1,149) = 1.14, *p* = 0.288, SECTRS, *F*(1,149) = 2.731, *p* = 0.101, NTSES, *F*(1,149) = 4.919, *p* = 0.028.

### 3.2 The overall effectiveness of the intervention on study outcomes

**Figure 1:**
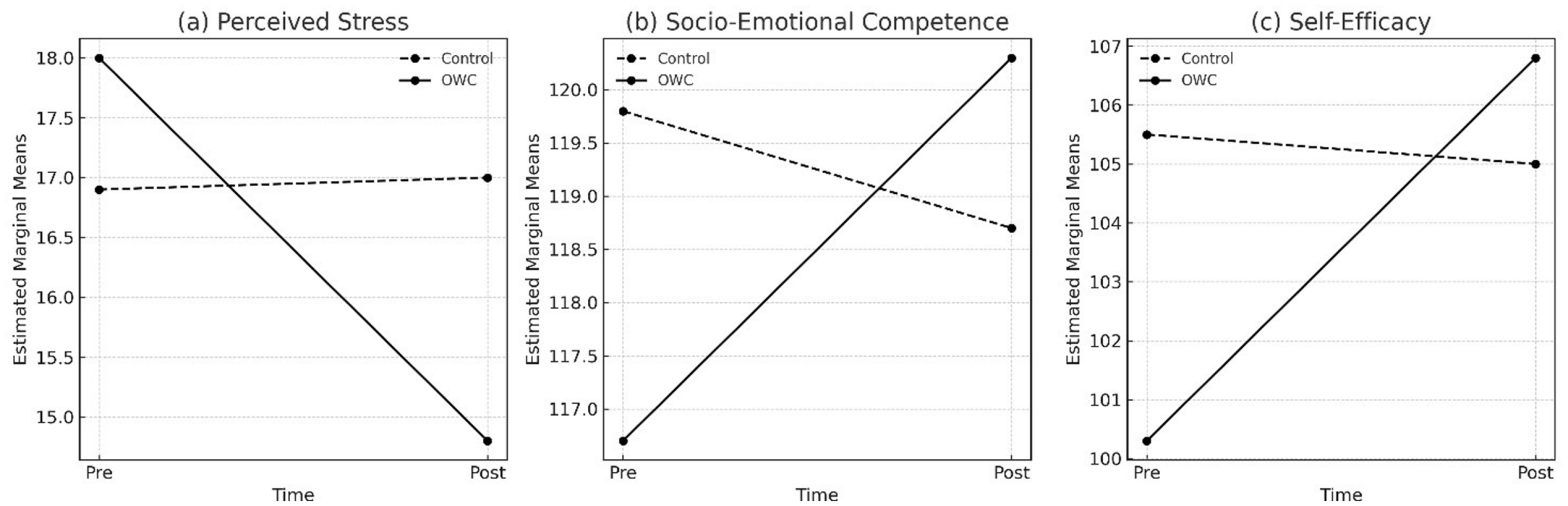
Estimated marginal means of perceived stress **(a)**, socio-emotional competence **(b)**, and self-efficacy **(c)** at pre- and post-test by group condition.

Results of the GLM analysis showed a significant Time*Group interaction, Wilks’s *λ* = 0.927, *F*(3,146) = 3.184, *p* = 0.011, *ր_p_*^2^ = 0.073. Univariate tests showed that this interaction was statistically significant for socio-emotional competence, *F*(1,148) = 5,029, *p* = 0.026, *ր_p_*^2^ = 0.033, and self-efficacy, *F*(1,148) = 6,318, *p* = 0.013, *ր_p_*^2^ = 0.041, and was marginally significant for perceived stress, F(1,148) = 7.187, *p* = 0.008, *ր_p_*^2^ = 0.046 (see Figure 1). The covariate years of teaching experience was not significant, suggesting that the effectiveness of the intervention was not influenced by teachers’ length of service, *p* > 0.05.

### 3.3 Mediation analysis

To examine whether the effects of the Online Wellbeing Course (OWC) on perceived stress were mediated by changes in socio-emotional competence and self-efficacy, we conducted a parallel multiple mediation analysis (PROCESS model 4; Hayes, 2022), controlling for years of teaching experience.

**Figure 2:**
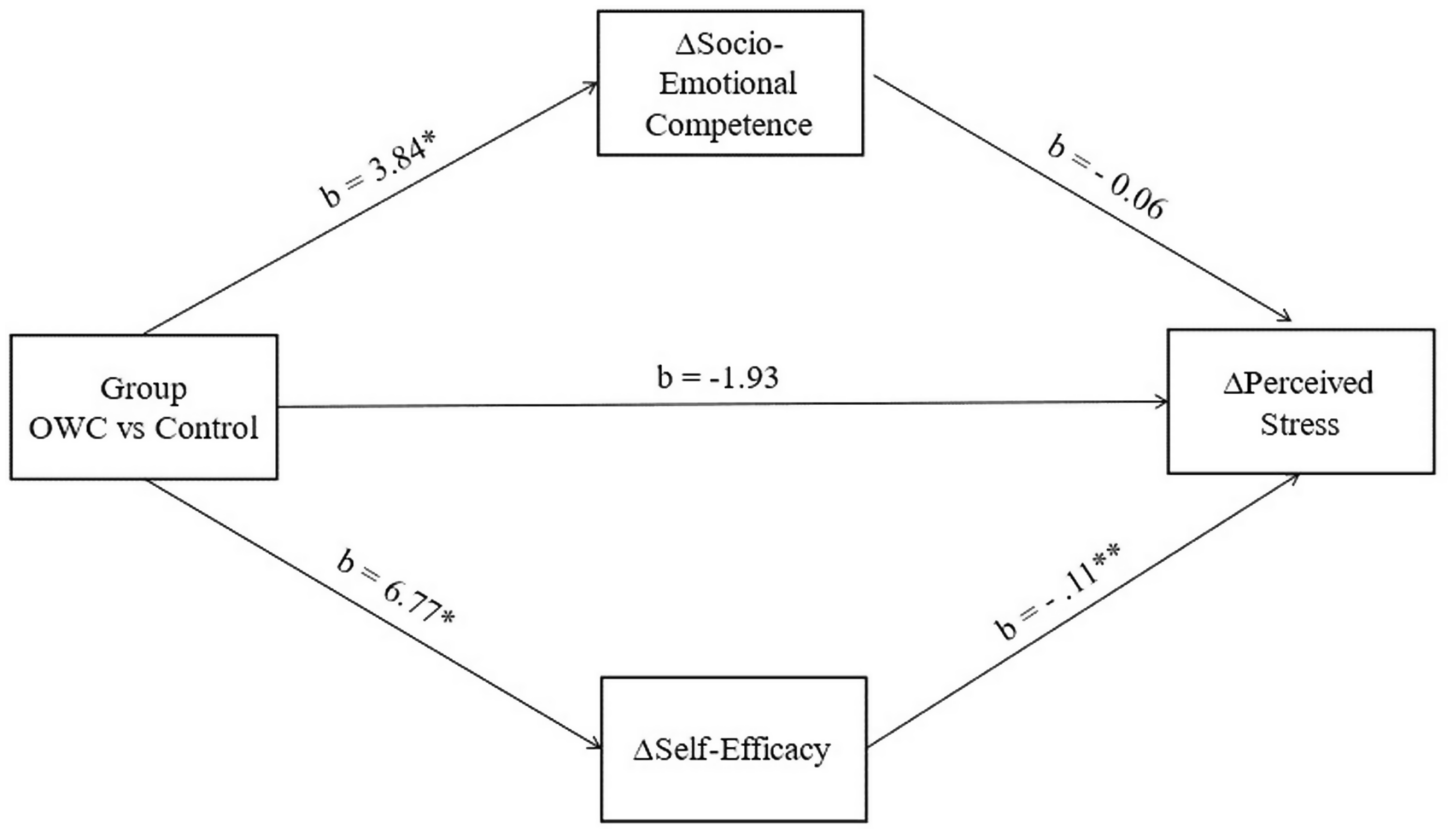
Parallel multiple mediation model testing the indirect effect of the Online Wellbeing Course (OWC) on changes in perceived stress through changes in socio-emotional competence and self-efficacy. Significant effects are indicated with asterisks: *p < 0.05, **p < 0.01.

As shown in Figure 2, the OWC intervention significantly predicted improvements in socio-emotional competence, *b* = 3.84, *p* = 0.026, 95% CI [0.46, 7.23], and self-efficacy, *b* = 6.77, *p* = 0.013, 95% CI [1.45, 12.09]. In the model predicting changes in perceived stress, increases in self-efficacy significantly predicted decreases in stress, *b* = −0.11, *p* = 0.002, 95% CI [−0.18, −0.04], indicating that teachers who gained more self-efficacy experienced greater reductions in perceived stress. In contrast, socio-emotional competence did not uniquely predict stress reduction, *b* = −0.06, *p* = 0.301, 95% CI [−0.16, 0.05].

The direct effect of the intervention on perceived stress was not statistically significant, *b* = −1.93, *p* = 0.070, 95% CI [−4.02, 0.16], indicating that the effect of the OWC on stress emerged primarily through indirect pathways. The total indirect effect of the OWC on stress was significant, indirect effect = −0.97, 95% CI [−2.09, −0.19]. Importantly, this effect was driven by the pathway through self-efficacy, indirect effect = − 0.75, 95% CI [−1.81, − 0.08], while the pathway through socio-emotional competence was not significant, indirect effect = − 0.22, 95% CI [−0.93, 0.28].

The analyses indicated that teaching experience, included as a covariate, significantly predicted changes in emotional competence, with more experienced teachers exhibiting greater improvements, *b* = 0.21, *p* = 0.010, 95% CI [0.05, 0.38]. In contrast, teaching experience was not a significant predictor of changes in self-efficacy, *b* = 0.04, *p* = 0.746, 95% CI [−0.21, 0.30], nor did it predict changes in stress after accounting for the mediators, *b* = −0.05, *p* = 0.285, 95% CI [−0.15, 0.05].

Taken together, these findings suggest that the OWC reduced perceived stress primarily through gains in teachers’ self-efficacy, independent of years of teaching experience, whereas socio-emotional competence did not uniquely account for stress reduction.

## 4 Discussion

The present study aimed to evaluate the effectiveness of an online wellbeing intervention for teachers (Online Wellbeing Course, OWC) in promoting their socio-emotional competence and self-efficacy, while also reducing levels of perceived stress. Findings partially confirmed our hypotheses. In line with H1, teachers in the experimental group reported significant improvements in socio-emotional competence and self-efficacy compared to the control group, together with a reduction in perceived stress. Moreover, consistent with H2, mediation analyses revealed that the effect of the OWC on perceived stress was fully mediated by increases in self-efficacy, whereas gains in socio-emotional competence did not directly impact on perceived stress reduction. When controlling for years of teaching experience, no significant effect emerged. Although teaching experience significantly predicted improvements in socio-emotional competence, it did not influence changes in self-efficacy or perceived stress. These findings will now be discussed in detail.

The pattern of a non-significant direct effect of the OWC on perceived stress in the presence of a significant indirect effect via self-efficacy is theoretically meaningful. This configuration reflects an ‘indirect-only’ mediation, indicating that the OWC did not reduce perceived stress per se, but did so insofar as it strengthened teachers’ beliefs in their capacity to manage professional demands. In this sense, self-efficacy operates as a proximal cognitive-motivational mechanism that translates participation in the intervention into changes in stress appraisal. Without such a modification in efficacy beliefs, exposure to the program alone appears insufficient to produce a direct decrease in perceived stress.

These results are consistent with previous literature highlighting the protective role of psychological resources in the school context. Specifically, they confirm that teachers’ self-efficacy constitutes a key factor in buffering the impact of occupational stressors (Schwarzer and Hallum, 2008; Zee and Koomen, 2016). A strong sense of efficacy sustains proactive coping strategies, perseverance under pressure, and adaptive classroom management, thereby preventing burnout and fostering a positive classroom climate. In this regard, our findings strengthen the evidence that interventions aimed at reinforcing teachers’ professional self-beliefs can directly contribute to lowering perceived stress and burnout. Teachers who strongly believe in their professional abilities are better able to manage work-related pressures and are less likely to experience emotional exhaustion and detachment. These findings are also consistent with prior evidence showing that targeted training interventions can significantly enhance teachers’ self-efficacy (Ansley et al., 2021), and extend this evidence by demonstrating that a digital, game-based intervention can strengthen efficacy beliefs in a relatively short timeframe, with measurable effects on perceived stress.

With respect to socio-emotional competences, although the program successfully enhanced these skills, they did not uniquely account for the reduction in participants’ perceived stress. One plausible explanation is that socio-emotional competences act mainly as long-term protective resources, supporting relational quality and work engagement (Ornaghi et al., 2023; Conte et al., 2025), but may not immediately affect teachers’ subjective appraisal of stress. It is important to note that in the present study socio-emotional competence was operationalized using a global composite self-report index. Although this approach captures a broad profile of teachers’ socio-emotional functioning, it may have limited the possibility of conducting a fine-grained analysis of the distinct sub-dimensions of the construct. In particular, this aggregate measurement may have obscured the temporal dynamics through which specific components, such as emotion regulation, exert their effects on perceived stress, potentially masking differential short-term versus longer-term impact patterns. Therefore, future studies are strongly recommended to examine the mediating role of distinct SEC sub-dimensions using marker-specific measures, rather than relying solely on composite scores. An additional explanation for why self-efficacy, but not socio-emotional competence, uniquely predicted reductions in perceived stress may be related to the specific design of the OWC intervention. The program was implemented as a gamified serious game, combining immersive scenarios, narrative avatars, quizzes, and progress tracking systems to engage teachers in active learning experiences (Cavioni et al., 2024). Such features likely provided teachers with repeated opportunities to practice problem-solving and classroom management strategies in a safe and controlled environment, thereby directly reinforcing their sense of personal competence and self-efficacy (Bandura, 1982; Skaalvik and Skaalvik, 2007). The immediate feedback and reward mechanisms embedded in the gamification elements further strengthened teachers’ perceptions of mastery, supporting the rapid consolidation of efficacy beliefs (Sailer et al., 2017; Soo and Cheng, 2022). In contrast, socio-emotional competences are primarily relational resources that develop and manifest through authentic interactions with students, colleagues, and the broader school environment (Jennings and Greenberg, 2009; Ornaghi et al., 2023). Although the OWC incorporated immersive, scenario-based, avatar-mediated interactions that realistically simulated classroom dilemmas, the transfer of socio-emotional competences from virtual environments to sustained real-life relational practices may require longer time frames and repeated *in vivo* application. Thus, the lack of immediate stress reduction linked to SEC may reflect not a deficiency of the online format itself, but rather the time needed for these relational skills to consolidate in daily school interactions. This interpretation is consistent with longitudinal research showing that socio-emotional competences protect against burnout and enhance work engagement over time rather than producing immediate stress relief (Conte et al., 2025). Moreover, because perceived stress reflects a subjective and cognitive appraisal of demands and resources (Lazarus and Folkman, 1984), it may be more immediately influenced by teachers’ efficacy beliefs than by socio-emotional competences, whose benefits are likely to emerge indirectly through improved classroom climate and professional relationships. Taken together, these considerations suggest that the OWC’s strong emphasis on task-oriented, feedback-rich, and self-reflective activities effectively boosted self-efficacy and thereby reduced perceived stress in the short term, whereas socio-emotional competence may function as long-term protective factors whose benefits require sustained interactional contexts to fully manifest.

Importantly, the effectiveness of the intervention did not vary as a function of teaching experience. Although more experienced teachers reported greater gains in socio-emotional competence, improvements in self-efficacy and the related reductions in perceived stress were consistent across career stages, in line with studies showing that years of teaching experience do not significantly predict perceived stress, whereas coping and emotion regulation strategies play a more decisive role in sustaining wellbeing (Messineo and Tosto, 2023). This suggests that digital wellbeing interventions such as the OWC may offer equitable benefits across different phases of the teaching profession, supporting both novice and veteran teachers in strengthening psychological resources.

Overall, these findings support theoretical perspectives recognizing the central role of teachers’ psychological resources (Jennings and Greenberg, 2009; Mansfield et al., 2016; Cavioni, 2025) and reinforce the idea that digital wellbeing interventions can be effective and accessible tools to promote teacher professional wellbeing in contemporary schools.

### 4.1 Digital wellbeing programs and serious games for teachers

Our outcomes are in line with very recent studies showing that online socio-emotional trainings and digital interventions can significantly reduce teachers’ stress and burnout, and enhance wellbeing (Ansley et al., 2021; Lillelien and Jensen, 2025; Romano et al., 2025). A distinctive and innovative element of this study was the use of a gamified digital program. Recent research (Liu et al., 2023; Cavioni et al., 2024) demonstrates that gamification enhances engagement and motivation in teacher training, providing interactive, immersive, and safe learning environments. Serious games, in particular, allow educators to practice coping and classroom management strategies within realistic simulations, experimenting freely without the risk of negative consequences (Dalgarno and Lee, 2010; Gower et al., 2025). Storytelling, narrative avatars, and reward systems foster curiosity, resilience, and persistence, thereby reinforcing the transfer of skills from virtual scenarios to professional practice.

Despite the growing adoption of digital tools in education, most gamified interventions have been developed for students rather than for teachers. Our findings suggest that serious games can fill this gap by directly supporting teachers’ socio-emotional skills, efficacy beliefs, and overall wellbeing. This represents a promising frontier in teacher professional development, combining accessibility, innovation, and personalization to meet the challenges of the contemporary educational context.

Our findings are consistent with previous qualitative evidence on the OWC. In a study based on teachers’ self-reported experiences, the course was perceived as useful for strengthening emotional awareness, stress regulation strategies, and relational competences (Cavioni et al., 2024). While such insights provided important exploratory knowledge on how teachers experienced the program, they relied exclusively on qualitative data. The present study extends this evidence by offering quantitative confirmation of the OWC’s effectiveness on validated measures of socio-emotional competence, self-efficacy, and perceived stress. Taken together, the two lines of research complement each other, showing both how teachers perceive the benefits of the intervention and how these translate into measurable psychological outcomes.

### 4.2 Limitations and future research directions

Despite the relevance of these findings, some limitations should be acknowledged. First, the quasi-experimental design does not allow for the same causal inferences as a randomized controlled trial, and the absence of follow-up data prevents us from evaluating the long-term sustainability of the observed effects. Second, exclusive reliance on self-report measures raises the possibility of bias due to social desirability or subjective perceptions. Third, with regard to the sample size, although it was adequate for the primary repeated-measures ANOVA (power >0.80), the post-hoc power analysis for the mediation model indicated only moderate statistical power (Monte Carlo power ≈ 0.70). Accordingly, the mediation findings should be considered as preliminary and informative of the direction of the proposed indirect effects, which should be further examined and confirmed in future studies with larger samples. In addition, although information was available on school level (preschool/elementary school versus middle/high school) and school location (urban versus rural) at the overall sample level, these variables were not included in the statistical analyses. Due to the randomization procedure (by school rather than by individual), the distribution of school levels was not balanced between the experimental and control groups, resulting in significant disparities in subgroup sizes. Under these conditions, stratified analyses would lack statistical robustness and would not allow for meaningful or interpretable conclusions. Further research using larger and more balanced samples is therefore warranted to investigate the potential moderating role of school grade and school context on the effectiveness of the intervention.

A further consideration is the cultural and contextual specificity of the sample, which comprised exclusively Italian teachers. Educational systems vary significantly across countries in terms of organizational demands, teacher training structures, and cultural norms regarding emotion expression and stress management. As such, the generalizability of the findings to other contexts remains limited. Cross-cultural replications and comparative studies are needed to establish the broader applicability of the intervention.

Future research should adopt longitudinal and multi-method designs (including observations, physiological indicators, and external ratings) to examine the mechanisms of change more comprehensively. It would also be useful to compare different intervention formats (online, blended, face-to-face) and to investigate the moderating role of contextual factors such as school level, teaching experience, and organizational support. Finally, further studies should explore in greater depth the contribution of gamification elements to teachers’ engagement and wellbeing, including through qualitative analyses.

### 4.3 Educational implications

From an educational perspective, the results have significant implications. They highlight the importance of embedding wellbeing programs within teacher professional development pathways, alongside disciplinary and instructional training. Promoting self-efficacy and socio-emotional competence not only reduces teachers’ stress but also strengthens instructional quality, classroom management, and student wellbeing, consistent with findings from Jennings and Greenberg (2009) and Zee and Koomen (2016).

The experience with the Online Wellbeing Course suggests that interactive digital training may represent a promising direction for supporting teachers’ professional wellbeing. Rather than acting as an add-on, this kind of intervention shows potential to reshape professional learning by cultivating teachers’ self-efficacy, emotional awareness, and adaptive strategies for handling stress. The simulation of authentic scenarios, combined with guided reflection, appears to help educators develop practices that extend beyond the game environment and into their daily routines. Embedding digital training programs within both pre-service and in-service teacher education could foster sustainable improvements in teacher self-efficacy and wellbeing (Täschner et al., 2025). For novice teachers, such programs may help build foundational coping skills and efficacy beliefs, thereby reducing vulnerability to early-career stress. For experienced teachers, they may serve as a means to counteract cumulative strain and bureaucratic overload, sustaining motivation and professional engagement across the career trajectory (Cavioni et al., 2024).

## Data Availability

The raw data supporting the conclusions of this article will be made available by the authors, without undue reservation.

## Footnotes

1
https://teachingtobe.eu/

## References

[ref1] Agyapong B. Obuobi-Donkor G. Burback L. Wei Y. (2022). Stress, burnout, anxiety and depression among teachers: a scoping review. Int. J. Environ. Res. Public Health 19:10706. doi: 10.3390/ijerph191710706, 36078422 PMC9518388

[ref2] Aloe A. M. Amo L. C. Shanahan M. E. (2014). Classroom management self-efficacy and burnout: a multivariate meta-analysis. Educ. Psychol. Rev. 26, 101–126. doi: 10.1007/s10648-013-9244-0

[ref3] Ansley B. M. Houchins D. E. Varjas K. Roach A. Patterson D. Hendrick R. (2021). The impact of an online stress intervention on burnout and teacher efficacy. Teach. Teach. Educ. 98:103251. doi: 10.1016/j.tate.2020.103251

[ref4] Avanzi L. Miglioretti M. Velasco V. Balducci C. Vecchio L. Fraccaroli F. . (2013). Cross-validation of the Norwegian teacher's self-efficacy scale (NTSES). Teach. Teach. Educ. 31, 69–78. doi: 10.1016/j.tate.2013.01.002, 41368200

[ref5] Bandura A. (1982). Self-efficacy mechanism in human agency. Am. Psychol. 37:122. doi: 10.1037/0003-066X.37.2.122

[ref6] Berg M. K. Talvio M. Hietajärvi L. Benítez I. Cavioni V. Conte E. . (2021). The development of teachers' and their students' social and emotional learning during the “learning to be project” in five European countries. Front. Psychol. 12:705336. doi: 10.3389/fpsyg.2021.705336 34484059 PMC8414967

[ref7] Bianchi R. Schonfeld I. S. (2016). Burnout is associated with a depressive cognitive style. Pers. Individ. Differ. 100, 1–5. doi: 10.1016/j.paid.2016.01.008

[ref8] Bottiani J. H. Duran C. A. K. Pas E. T. Bradshaw C. P. (2019). Teacher stress and burnout in urban middle schools: associations with job demands, resources, and effective classroom practices. J. Sch. Psychol. 77, 36–51. doi: 10.1016/j.jsp.2019.10.002, 31837727

[ref9] Brackett M. A. (2019). Permission to feel. The power of emotional intelligence to achieve well-being and success. New York: Celadon Books.

[ref10] Brackett M. A. Palomera R. Mojsa-Kaja J. Reyes M. R. Salovey P. (2011). Emotion-regulation ability, burnout, and job satisfaction among British secondary-school teachers. Psychol. Sch. 47, 406–417. doi: 10.1002/pits.20478, 41370133

[ref11] Brewer E. W. Shapard L. (2004). Employee burnout: a meta-analysis of the relationship between age or years of experience. Hum. Resour. Dev. Rev. 3, 102–123. doi: 10.1177/1534484304263335

[ref12] Capone V. Joshanloo M. Sang-Ah Park M. (2019). Burnout, depression, efficacy beliefs, and work-related variables among school teachers. Int. J. Educ. Res. 95, 97–108. doi: 10.1016/j.ijer.2019.02.001

[ref13] Carroll A. Forrest K. Sanders-O’Connor E. Flynn L. Bower J. M. Fynes-Clinton S. . (2022). Teacher stress and burnout in Australia: examining the role of intrapersonal and environmental factors. Soc. Psychol. Educ. 25, 441–469. doi: 10.1007/s11218-022-09686-7, 35233183 PMC8874312

[ref14] Cavioni V. (2025). Fostering teachers’ mental health: Evidence from theory, research, and practice. Cham: Springer Nature.

[ref15] Cavioni V. Conte E. Ornaghi V. (2024). Promoting teachers’ wellbeing through a serious game intervention: a qualitative exploration of teachers’ experiences. Front. Psychol. 15:1339242. doi: 10.3389/fpsyg.2024.1339242, 38601821 PMC11004468

[ref16] Cavioni V. Grazzani I. Ornaghi V. Agliati A. Gandellini S. Cefai C. . (2023a). A multi-component curriculum to promote teachers’ mental health: findings from the PROMEHS program. Int. J. Emot. Educ. 15, 34–52. doi: 10.3389/fpsyg.2023.1229653

[ref17] Cavioni V. Toto G. Ornaghi V. (2023b). Portraits of pre-service special education teachers: perspectives on well-being and its association with self-efficacy and commitment to work. Int. J. Emot. Educ. 15, 21–36. doi: 10.56300/VHRV8364

[ref18] Cervellione B. Lombardo E. M. C. Calogero I. (2025). Psychological resources and interventions for teachers' emotional competence and well-being: a systematic review. Front. Psychol. 16:1640968. doi: 10.3389/fpsyg.2025.1640968, 40893859 PMC12394466

[ref19] Cohen S. Kamarck T. Mermelstein R. (1983). A global measure of perceived stress. J. Health Soc. Behav. 24, 385–396. doi: 10.2307/2136404, 6668417

[ref20] Collie R. J. Perry N. E. Martin A. J. (2017). “School context and educational system factors impacting educator stress” in Educator stress: An occupational health perspective. eds. McIntyre T. M. McIntyre S. E. Francis D. J. (New York, NY: Springer).

[ref21] Collie R. J. Shapka J. D. Perry N. E. (2012). School climate and social–emotional learning: predicting teacher stress, job satisfaction, and teaching efficacy. J. Educ. Psychol. 104:1189. doi: 10.1037/a0029356

[ref22] Conte E. Cavioni V. Ornaghi V. (2024). Exploring stress factors and coping strategies in Italian teachers after COVID-19: evidence from qualitative data. Educ. Sci. 14:152. doi: 10.3390/educsci14020152

[ref23] Conte E. Farina E. Pepe A. Cavioni V. Ornaghi V. (2025). The longitudinal effects of teachers’ socio-emotional competences on stress and burnout. J. Exp. Educ. 1-15, 1–15. doi: 10.1080/00220973.2025.2518231, 41307611

[ref24] Cooper C. Travers C. (2012). Teachers under pressure: Stress in the teaching profession. London: Routledge.

[ref25] Dalgarno B. Lee M. J. (2010). What are the learning affordances of 3-D virtual environments? Br. J. Educ. Technol. 41, 10–32. doi: 10.1111/j.1467-8535.2009.01038.x

[ref26] Durlak J. A. Domitrovich C. E. Weissberg R. P. Gullotta T. P. (2025). Handbook of social and emotional learning: Research and practice. 2nd Edn. New York, NY: Guilford Press.

[ref27] Fossati A. (2010). Italian translation of the perceived stress scale. Available online at: http://www.pensierocritico.eu/files/Italian_PSS_10_with_info.pdf

[ref28] Gavish B. Friedman I. A. (2010). Novice teacher’s experience of teaching: a dynamic aspect of burnout. Soc. Psychol. Educ. 13, 141–167. doi: 10.1007/s11218-009-9108-0

[ref29] George D. Mallery M. (2010). SPSS for windows step by step: A simple guide and reference, 17.0 update. 10a Edn. Boston: Pearson.

[ref30] Geraci A. Di Domenico L. Inguglia C. D’Amico A. (2023). Teachers’ emotional intelligence, burnout, work engagement, and self-efficacy during COVID-19 lockdown. Behav. Sci. 13:296. doi: 10.3390/bs13040296, 37102810 PMC10135634

[ref31] Gooden C. Zelkowski J. Smith F. A. (2023). A systematic literature review on factors of stress, burnout and job satisfaction of secondary grades teachers at time of professional crisis. Clear. House 96, 162–171. doi: 10.1080/00098655.2023.2238880

[ref32] Gower I. F. Lee D. Palmer E. (2025). “Immersive opportunities: a systematic review of virtual reality in the classroom” in Risks and opportunities in using educational technologies (Singapore: Springer), 87–115.

[ref33] Grazzani I. Martinsone B. Simoes C. Cavioni V. Conte E. Ornaghi V. . (2024). Assessing teachers’ social and emotional competence: the validation of SECTRS in Italy, Latvia, and Portugal. Int. J. Emot. Educ. 16, 70–87. doi: 10.56300/QIAN8168

[ref9001] Hayes A. F. (2022). Introduction to mediation, moderation, and conditional process analysis: A regression-based approach (3rd ed.). New York: Guilford Press.

[ref34] Hettinger K. Lazarides R. Schiefele U. (2024). Longitudinal relations between teacher self-efficacy and student motivation through matching characteristics of perceived teaching practice. Eur. J. Psychol. Educ. 39, 1299–1325. doi: 10.1007/s10212-023-00744-y

[ref35] Jennings P. A. Greenberg M. T. (2009). The prosocial classroom: teacher social and emotional competence in relation to student and classroom outcomes. Rev. Educ. Res. 79, 491–525. doi: 10.3102/0034654308325693

[ref36] Jentsch A. Hoferichter F. Blömeke S. König J. Kaiser G. (2023). Investigating teachers’ job satisfaction, stress and working environment: the roles of self-efficacy and school leadership. Psychol. Sch. 60, 679–690. doi: 10.1002/pits.22788

[ref37] Kalogiannakis M. Papadakis S. Zourmpakis A. I. (2021). Gamification in science education. A systematic review of the literature. Educ. Sci. 11:22. doi: 10.3390/educsci11010022

[ref38] Kapp K. M. (2012). The gamification of learning and instruction: Game-based methods and strategies for training and education. San Francisco, CA: Pfeiffer.

[ref39] Kariou A. Koutsimani P. Montgomery A. Lainidi O. (2021). Emotional labor and burnout among teachers: a systematic review. Int. J. Environ. Res. Public Health 18:12760. doi: 10.3390/ijerph182312760, 34886485 PMC8657663

[ref40] Katsarou E. Chatzipanagiotou P. Sougari A. M. (2023). A systematic review on teachers’ well-being in the COVID-19 era. Educ. Sci. 13:927. doi: 10.3390/educsci13090927

[ref41] Kyriacou C. (2001). Teacher stress: directions for future research. Educ. Rev. 53, 27–35. doi: 10.1080/00131910120033628

[ref42] Kyriacou C. (2011). “Teacher stress: from prevalence to resilience” in Handbook of stress in the occupations. eds. Langan-Fox J. Cooper C. L. (Cheltenham: Edward Elgar), 161–173.

[ref43] Lazarus R. S. Folkman S. (1984). Stress, appraisal, and coping. New York: Springer.

[ref44] Levante A. Petrocchi S. Bianco F. Castelli I. Lecciso F. (2023). Teachers during the COVID-19 era: the mediation role played by mentalizing ability on the relationship between depressive symptoms, anxious trait, and job burnout. Int. J. Environ. Res. Public Health 20:859. doi: 10.3390/ijerph20010859, 36613181 PMC9820251

[ref45] Lillelien K. Jensen M. T. (2025). Digital and digitized interventions for teachers’ professional well-being: a systematic review of work engagement and burnout using the job demands–resources theory. Educ. Sci. 15:799. doi: 10.3390/educsci15070799

[ref46] Liu T. Oubibi M. Zhou Y. Fute A. (2023). Research on online teachers’ training based on the gamification design: a survey analysis of primary and secondary school teachers. Heliyon 9:e15053. doi: 10.1016/j.heliyon.2023.e15053, 37082639 PMC10112028

[ref47] Lizana P. A. Vega-Fernadez G. Gomez-Bruton A. Leyton B. Lera L. (2021). Impact of the COVID-19 pandemic on teacher quality of life: a longitudinal study from before and during the health crisis. Int. J. Environ. Res. Public Health 18:3764. doi: 10.3390/ijerph18073764, 33916544 PMC8038473

[ref48] Mansfield C. F. (2020). Cultivating teacher resilience: International approaches, applications and impact. Singapore: Springer Nature, 307.

[ref49] Mansfield C. F. Beltman S. Broadley T. Weatherby-Fell N. (2016). Building resilience in teacher education: an evidenced informed framework. Teach. Teach. Educ. 54, 77–87. doi: 10.1016/j.tate.2015.11.016

[ref50] Martel-Santana A. Martín-del-Pozo M. (2025). A usability evaluation of a serious game for tackling bullying and cyberbullying in primary education by pre-service teachers. Technol. Knowl. Learn. 30, 2035–2079. doi: 10.1007/s10758-025-09850-w

[ref51] Maslach C. Jackson S. E. (1981). The measurement of experienced burnout. J. Organ. Behav. 2, 99–113. doi: 10.1002/job.4030020205

[ref52] Maslach C. Schaufeli W. B. Leiter M. P. (2001). Job burnout. Annu. Rev. Psychol. 52, 397–422. doi: 10.1146/annurev.psych.52.1.397, 11148311

[ref54] McCormick J. Barnett K. (2011). Teachers’ attributions for stress and their relationships with burnout. Int. J. Educ. Manag. 25, 278–293. doi: 10.1108/09513541111120114

[ref55] Mérida-López S. Extremera N. (2017). Emotional intelligence and teacher burnout: a systematic review. Int. J. Educ. Res. 85, 121–130. doi: 10.1016/j.ijer.2017.07.006

[ref56] Messineo L. Tosto C. (2023). Perceived stress and affective experience in Italian teachers during the COVID-19 pandemic: correlation with coping and emotion regulation strategies. Eur. J. Psychol. Educ. 38, 1271–1293. doi: 10.1007/s10212-022-00661-6, 40479454 PMC9734932

[ref57] Mijakoski D. Cheptea D. Marca S. C. Shoman Y. Caglayan C. Bugge M. D. . (2022). Determinants of burnout among teachers: a systematic review of longitudinal studies. Int. J. Environ. Res. Public Health 19:5776. doi: 10.3390/ijerph19095776, 35565168 PMC9104901

[ref58] Nwoko J. C. Emeto T. I. Malau-Aduli A. E. Malau-Aduli B. S. (2023). A systematic review of the factors that influence teachers’ occupational wellbeing. Int. J. Environ. Res. Public Health 20:6070. doi: 10.3390/ijerph20126070, 37372657 PMC10298565

[ref59] Ornaghi V. Conte E. Cavioni V. Farina A. Pepe A. (2023). The role of teachers’ socio-emotional competence in reducing burnout through increased work engagement. Front. Psychol. 14:1295365. doi: 10.3389/fpsyg.2023.1295365, 38022976 PMC10644694

[ref60] Pekrun R. (2006). The control-value theory of achievement emotions: assumptions, corollaries, and implications for educational research and practice. Educ. Psychol. Rev. 18, 315–341. doi: 10.1007/s10648-006-9029-9

[ref61] Pekrun R. (2024). Control-value theory: from achievement emotion to a general theory of human emotions. Educ. Psychol. Rev. 36, 1–36. doi: 10.1007/s10648-024-09909-7

[ref62] Pellerone M. (2021). Self-perceived instructional competence, self-efficacy and burnout during the covid-19 pandemic: a study of a group of Italian school teachers. Eur. J. Investigat. Health Psychol. Educ. 11, 496–512. doi: 10.3390/ejihpe11020035, 34708818 PMC8314360

[ref63] Peterson C. Maier S. F. Seligman M. E. P. (1993). Learned helplessness: A theory for the age of personal control. Oxford: Oxford University Press.

[ref64] Romano G. V. S. Bazán P. R. Sato J. R. da Cruz N. V. C. J. Pacifico E. D. S. Lacerda S. S. . (2025). Mental health outcomes following a four-week online training on social emotional and ethical learning for public school teachers. Sci. Rep. 15:9179. doi: 10.1038/s41598-025-91967-0, 40097467 PMC11914406

[ref65] Rooney P. (2012). A theoretical framework for serious game design: exploring pedagogy, play and fidelity and their implications for the design process. Int. J. Game-Based Learn. 2, 41–60. doi: 10.4018/ijgbl.2012100103

[ref66] Sacré M. Ries N. Wolf K. Kunter M. (2023). Teachers’ well-being and their teaching quality during the COVID-19 pandemic: a retrospective study. Front. Educ. 8:1136940. doi: 10.3389/feduc.2023.1136940

[ref67] Sailer M. Hense J. U. Mayr S. K. Mandl H. (2017). How gamification motivates: an experimental study of the effects of specific game design elements on psychological need satisfaction. Comput. Hum. Behav. 69, 371–380. doi: 10.1016/j.chb.2016.12.033

[ref68] Schaufeli W. B. Buunk B. P. (2004). “Burnout: an overview of 25 years of research and theorizing” in The handbook ofWork and Health Psychology. eds. Schabracq M. J. Winnubst J. A. M. Cooper C. L.. 2nd ed (Hoboken, NJ: Wiley), 383–425.

[ref69] Schwarzer R. Hallum S. (2008). Perceived teacher self-efficacy as a predictor of job stress and burnout: mediation analyses. Appl. Psychol. 57, 152–171. doi: 10.1111/j.1464-0597.2008.00359.x

[ref70] Seaborn K. Fels D. I. (2015). Gamification in theory and action: a survey. Int. J. Hum.-Comput. Stud. 74, 14–31. doi: 10.1016/j.ijhcs.2014.09.006

[ref71] Shute V. J. D’Mello S. Baker R. Cho K. Bosch N. Ocumpaugh J. . (2015a). Modeling how incoming knowledge, persistence, affective states, and in-game progress influence student learning from an educational game. Comput. Educ. 86, 224–235. doi: 10.1016/j.compedu.2015.08.001

[ref72] Shute V. J. Ventura M. Ke F. (2015b). The power of play: the effects of portal 2 and Lumosity on cognitive and noncognitive skills. Comput. Educ. 80, 58–67. doi: 10.1016/j.compedu.2014.08.013

[ref73] Skaalvik E. M. Skaalvik S. (2007). Dimensions of teacher self-efficacy and relations with strain factors, perceived collective teacher efficacy, and teacher burnout. J. Educ. Psychol. 99, 611–625. doi: 10.1037/0022-0663.99.3.611

[ref74] Soo C. Cheng J. L. A. (2022). The psychology of rewards in digital game-based learning: a comprehensive review. J. Cogn. Sci. Hum. Dev. 8:1. doi: 10.33736/jcshd.4131.2022

[ref75] Swacha J. (2021). State of research on gamification in education: a bibliometric survey. Educ. Sci. 11:69. doi: 10.3390/educsci11020069

[ref76] Täschner J. Dicke T. Reinhold S. Holzberger D. (2025). “Yes, I can!” a systematic review and meta-analysis of intervention studies promoting teacher self-efficacy. Rev. Educ. Res. 95, 3–52. doi: 10.3102/00346543231221499

[ref77] Tom K. M. (2012). Measurement of teachers’ social-emotional competence: development of the social-emotional competence teacher rating scale (doctoral dissertation). Available online at: https://scholarsbank.uoregon.edu/xmlui/bitstream/handle/1794/12351/Tom_oregon_0171A_10250.pdf?sequence=1&isAllowed=y

[ref78] Travers C. J. Cooper C. L. (1996). Teachers under pressure: Stress in the teaching profession. New York: Psychology Press.

[ref79] Van Droogenbroeck F. Spruyt B. Vanroelen C. (2014). Burnout among senior teachers: investigating the role of workload and interpersonal relationships at work. Teach. Teach. Educ. 43, 99–109. doi: 10.1016/j.tate.2014.07.005

[ref81] Zagni B. Pellegrino G. Ianes D. Scrimin S. (2025). Unraveling teacher stress: a cumulative model of risks and protective factors in Italian schools. Int. J. Educ. Res. 131, 102603–102614. doi: 10.1016/j.ijer.2025.102603, 41368200

[ref82] Zee M. Koomen H. M. Y. (2016). Teacher self-efficacy and its effects on classroom processes, student academic adjustment, and teacher well-being: a synthesis of 40 years of research. Rev. Educ. Res. 86, 981–1015. doi: 10.3102/0034654315626801

[ref83] Zhou S. Slemp G. R. Vella-Brodrick D. A. (2024). Factors associated with teacher wellbeing: a meta-analysis. Educ. Psychol. Rev. 36, 1–48. doi: 10.1007/s10648-024-09886-x, 41369767

